# *nu444* is a novel allele of *pkc-1* in *C. elegans*

**DOI:** 10.17912/W2Z59X

**Published:** 2017-05-18

**Authors:** Han Wang, Derek Sieburth

**Affiliations:** 1 Zilkha Neurogenetic Institute, Keck School of Medicine, University of Southern California, Los Angeles, CA 90033, USA; 2 Present Address: Division of Biology and Biological Engineering and Howard Hughes Medical Institute, California Institute of Technology, Pasadena, CA 91125, USA

**Figure 1.**
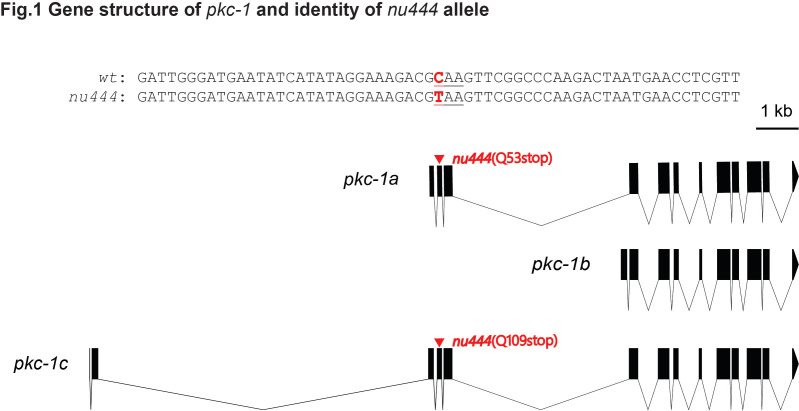


## Description

Here, we report *nu444* as a novel allele of the gene *pkc-1* that encodes the protein kinase C-1 in C. elegans. The *nu444* allele was originally isolated from a forward genetic screen for mutants that suppressed the “Hic” (Hypersensitivity to Inhibitors of Cholinesterase) phenotype of *dgk-1*(*nu62*) mutants, which had increased acetylcholine release at the neuromuscular junction (Sieburth et al., 2007). In this screen, several genes that are important for neuropeptide secretion were recovered, including *pkc-1*(*nu448*) (Sieburth et al., 2007) and *ric-7*(*nu447*) (Hao et al., 2012). Sanger sequencing of the exons and exon-intron junctions of the *pkc-1* locus revealed that *nu444* had a nonsense mutation (C to T, in the coding strand of *pkc-1*, with left flanking sequence: 5’-GATGAATATCATATAGGAAAGACG-3’ and right flanking sequence: 5’- AAGTTCGGCCCAAGACTAATGAACC-3’) in an early exon that is only present in pkc1a and pkc-1c isoforms (Fig.1). Thus, *pkc-1*(*nu444*) allele is probably a null allele for both *pkc-1*a (Q53stop) and *pkc-1*c (Q109stop), but presumably does not affect *pkc-1*b.​

## Reagents

KP1939 *pkc-1*(*nu444*) V; *dgk-1* (*nu62*) X

OJ580 *pkc-1*(*nu444*) V
